# Comparison between open-angle glaucoma and angle-closure glaucoma regarding the short-term optic disc vessel density changes after trabeculectomy

**DOI:** 10.1007/s10103-023-03907-x

**Published:** 2023-10-28

**Authors:** Nermien Salah El-Dien Mohammed El-Haddad, Adel Abd Elwahab, Sawssan Shalaby, Mona Mohammad Aly Farag, Mohammd Alkassaby, Sanaa Ahmed, Shrief Shawky

**Affiliations:** 1 Faculty of Medicine, Al-Azhar University, Cairo, Egypt; 2 Cairo, Egypt; 3 Al-Zharaa University Hospital, Al-Azhar University, Cairo, Egypt; 4 Faculty of Medicine, Armed Force Medicine Collage, Cairo, Egypt

**Keywords:** Angle-closure glaucoma, Open-angle glaucoma, Optic disc vessel density, OCT-A

## Abstract

To compare the microvasculature of the optic disc in open-angle glaucoma (OAG) and angle-closure glaucoma (ACG) after trabeculectomy. This study included 34 patients divided into two groups based on the angle: (1) the OAG Group, which included 24 eyes from 24 patients, and (2) the ACG Group, which included ten eyes from 10 patients. All patients were subjected to comprehensive ophthalmic examinations. It included best-corrected visual acuity (BCVA), Goldmann applanation tonometry, gonioscopy, slit-lamp biomicroscopy, dilated fundus examination, and stereoscopic examination of the optic disc. The central corneal thickness was measured using a Nidek AL scan optical biometer. The visual field was evaluated by standard automated perimetry using Humphrey Field Analyzer (24–2 Swedish interactive threshold algorithm; Carl-Zeiss Meditec, Dublin, CA). Moreover, optical coherence tomography angiography (OCT-A) was performed utilizing the RTVue XR Avanti scanner (Optovue Inc., Fremont, CA, USA) preoperatively as well as 1 month after surgery. There was a statistically significant increase in optic disc vessel density (VD) in the whole image, radial peripapillary, inferior, superior, temporal, and nasal quadrant in OAG after surgery. Regarding the change in VD after surgery in ACG patients, there was a statistically significant increase in optic disc VD in the whole image, radial peripapillary, inferior, superior, temporal, and nasal quadrant in ACG after surgery.

Regarding the percentage of VD change following trabeculectomy surgery, there were highly significant differences between the two groups for the whole image, radial peripapillary, inferior quadrant, and temporal quadrant VD. Statistically significant changes were also observed in the superior and nasal quadrants of VD. However, no significant change in VD was detected within the disc. In conclusion, the optic disc VD improved after the reduction of intraocular pressure (IOP) by surgery in both OAG and ACG. However, ACG demonstrated a significant improvement than the OAG.

## Introduction

Glaucoma is a leading cause of permanent blindness in both developed and developing countries. It is an optic neuropathy characterized by retinal ganglion cell axon degeneration, resulting in visual field loss due to progressive optic nerve damage [1].

There are several subtypes of glaucoma; in OAG, these eyes are characterized by an open angle on gonioscopy and glaucomatous visual field impairment. ACG is the angle of the anterior chamber that is occludable during gonioscopy in three or more quadrants [2].

Increased intraocular pressure (IOP) is the primary factor in the pathophysiology of glaucoma. However, it has been demonstrated that disruptions in ocular blood flow play a role in the pathophysiology of glaucoma. Several imaging techniques have been used to evaluate ocular perfusion in glaucoma, such as the Heidelberg retina flowmeter, laser speckle flowgraphy (LSFG), color Doppler imaging, fluorescein angiography (FA), and laser Doppler flowmetry (LDF) [3].

A reduction in papillary VD has been reported in eyes with glaucoma. In addition, a possible correlation has been hypothesized between the diameter of the retinal arterioles and optic nerve damage [4].

Optical coherence tomography angiography (OCT-A) is a rapid, non-invasive, reliable diagnostic modality that can assess the perfusion of different retinal layers, the optic disc, and the peripapillary vascular density [5].

In this study, the optic disc VD was compared after IOP reduction via trabeculectomy in both OAG and ACG.

## Patients and method

This study was conducted at the Al-Zharaa University Hospital. It included 34 patients divided into two groups based on the angle: (1) the open-angle (OAG) group, which consisted of 24 eyes from 24 patients and (2) the closed-angle (ACG) group, which included 10 eyes from 10 patients. The study was conducted in accordance with the principles of the Helsinki Declaration. Informed written consent was obtained from all patients.

Inclusion criteria included (1) open-angle or closed-angle glaucoma, (2) indication of surgery (progression of disease despite complete medical treatment, patient non-compliance), (3) clear optical media, and (4) absence of secondary glaucoma.

Exclusion criteria included (1) history of systemic diseases, (2) systemic drugs that affect blood vessels (e.g., dopamine antagonist), (3) any media opacity, (4) refraction more than − 6D, (5) patient not indicated for surgery, (6) history of previous ocular surgery or trauma, (7) smoking and alcohol intake, and (8) postoperative IOP less than 6 mmHg and hypotensive maculopathy that both led to patient exclusion from follow-up.

All patients were subjected to comprehensive ophthalmic examinations. It included best-corrected visual acuity (BCVA), Goldmann applanation tonometry, gonioscopy, slit-lamp biomicroscopy, dilated fundus examination, and stereoscopic examination of the optic disc. The central corneal thickness was measured using a Nidek AL scan optical biometer. The visual field was evaluated by standard automated perimetry using Humphrey Field Analyzer (24–2 SITA; Carl-Zeiss Meditec, Dublin, CA). Additionally, OCT-A was performed using the RTVueXR Avanti scanner (Optovue AngioVue System, software ReVue XR version 2017.1.0.151, Optovue Inc., Fremont, CA, USA)) preoperatively as well as 1 month after surgery.

### Surgical technique

The trabeculectomy was performed under local anesthesia. A partial thickness traction suture was performed on the clear corneal periphery. The superior sclera was exposed through a fornix-based conjunctival peritomy. Hemostasis was achieved through the use of gentle cautery. In the superior sclera, a partial thickness triangular scleral flap was created. The scleral flap was dissected forward until the grayish-blue zone at the limbus was exposed.

Before the ostomy, a paracentesis was performed on the peripheral clear cornea. The ostomy was then created with a blade and expanded with Vannas scissors. A peripheral iridectomy has been performed. The scleral flap was closed using 10–0 nylon sutures. Then, the peritomy of the conjunctiva was closed with absorbable sutures.

### Optical coherence tomography angiography measurements

The OCT-A device used in this study was Avanti RTVue-XR with the AngioVue software (version 2017.1.0.151; Optavue, Inc.) using the SSADA algorithm. The motion artifacts and fixation changes were avoided by taking two horizontal and vertical volume scans. The AngioVue software was used to obtain automated VD measurements. OCT-A images of the retina were captured using a 4.5 × 4.5 mm disc. VD is automatically determined by dividing images received from the optical disc into previously defined regions. In order to evaluate radial peripapillary capillary (RPC) vessel density, images are automatically segmented by the OCT-A device in the peripapillary region. The image quality (signal strength > 7/10) and absence of artifacts were evaluated.

OCT-A was performed 1 week prior to surgery and 1 month postoperatively. The percentage of VD change between the two groups was compared. One physician performed the procedure between 9 and 11 am to avoid diurnal variations.

## Results

There was no statistical difference between the two study groups regarding age, sex, and laterality, with *P*-values of (0.809, 0.754, and 0.329), respectively (Table 1).

**Table 1:** The demographic data of open-angle and angle-closure glaucoma

		Open angle	Closed angle	Test value	*P*-value	Sig.
		No. = 24	No. = 10			
Gender	Female	6 (25.0%)	2 (20.0%)	0.098*	0.754	NS
	Male	18 (75.0%)	8 (80.0%)			
Age (years)	Mean ± SD	56.25 ± 11.37	57.20 ± 7.22	− 0.243•	0.809	NS
	Range	39–76	47–68			
Laterality	Right	10 (41.7%)	6 (60.0%)	0.952*	0.329	NS
	Left	14 (58.3%)	4 (40.0%)			

*P*-value > 0.05: non significant; *P*-value < 0.05: significant; *P*-value < 0.01: highly significant

*Chi-square test

•Independent *t*-test

In addition, there were no statistically significant differences between the two groups in terms of the preoperative IOP, postoperative IOP, percent of IOP reduction after surgery, visual acuity (VA), cub/disc ratio (C/D), mean deviation (MD), and central corneal thickness (CCT). There was a statistically significant difference between both groups as regards the total retinal nerve fiber layer (TRNFL) (Table 2).

**Table 2:** Comparison between open-angle and closed-angle glaucoma groups as regards the examination parameters

Examination parameters		Open angle	Closed angle	Test value	*P*-value	Sig.
		No. = 24	No. = 10			
IOP pre	Mean ± SD	26.92 ± 3.11	28.60 ± 3.44	− 1.396•	0.172	NS
	Range	24–34	23–33			
IOP post	Mean ± SD	11.25 ± 1.96	11.40 ± 1.26	− 0.222•	0.826	NS
	Range	8–15	10–13			
IOP % of reduction	Mean ± SD	58.24 ± 5.25	59.38 ± 8.60	− 0.478•	0.636	NS
	Range	50–66.67	43.48–65.52			
VA	Mean ± SD	0.88 ± 0.13	0.80 ± 0.12	1.619•	0.115	NS
	Range	0.6–1	0.7–1			
C/D	Mean ± SD	0.63 ± 0.10	0.62 ± 0.12	0.321•	0.750	NS
	Range	0.5–0.8	0.5–0.8			
CCT	Mean ± SD	514.08 ± 42.15	537.60 ± 30.26	− 1.595•	0.121	NS
	Range	452–605	495–580			
MD	Mean ± SD	-10.33 ± 2.30	− 10.08 ± 1.47	− 0.309•	0.759	NS
	Range	− 14.8 to − 6.9	− 12.7 to − 8.6			
Total RNFL	Mean ± SD	75.42 ± 11.12	64.80 ± 4.24	2.910•	0.007	HS
	Range	61–98	59–71			

*P*-value > 0.05: non significant; *P*-value < 0.05: significant; *P*-value < 0.01: highly significant

•Independent *t*-test

**Fig. 1:**
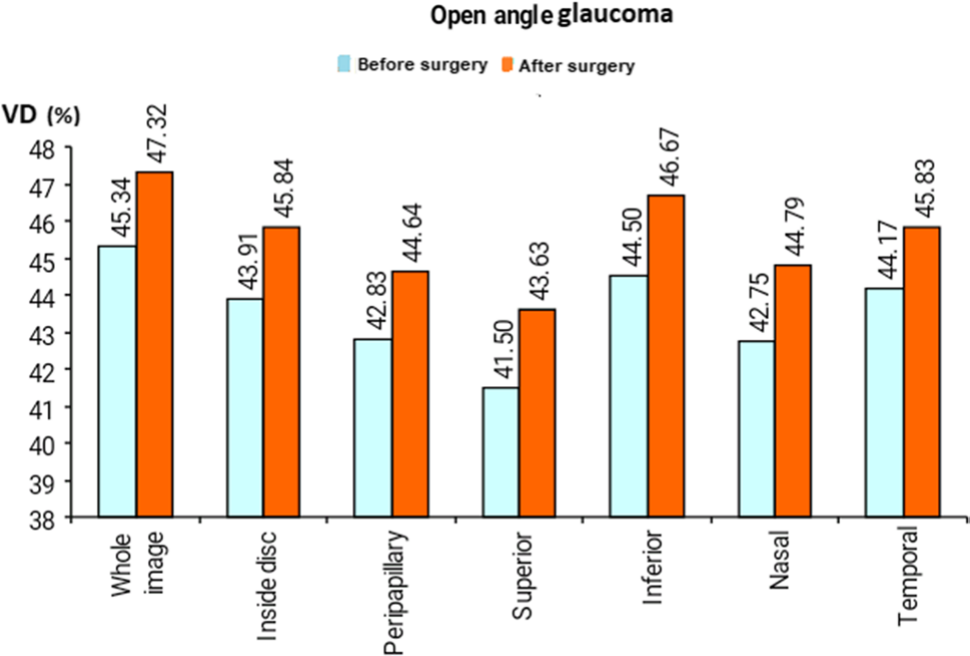
Comparison of optic disc vessel density (VD) before and after trabeculectomy in open-angle glaucoma (OAG)

There was a statistically significant increase in optic disc VD in the whole image, radial peripapillary, inferior, superior, temporal, and nasal quadrant in OAG after surgery (Fig. 1 and Table 3).

**Table 3:** The optic disc vessel density after trabeculectomy in open-angle glaucoma

Vessel density %		Open angle		Test value	*P*-value	Sig.
		Before surgery	After surgery			
Whole image	Mean ± SD	45.34 ± 3.70	47.32 ± 3.00	− 2.475•	0.021	S
	Range	38.3–50.9	39.8–50.2			
Inside disc	Mean ± SD	43.91 ± 2.64	45.84 ± 3.27	− 2.275•	0.033	S
	Range	37.7–47.9	41.8–51.9			
Peripapillary	Mean ± SD	42.83 ± 2.82	44.64 ± 5.02	− 2.331•	0.029	S
	Range	37.1–46.3	38.5–52			
Superior	Mean ± SD	41.50 ± 4.87	43.63 ± 6.91	− 2.470•	0.021	S
	Range	31–49	33–54			
Inferior	Mean ± SD	44.50 ± 4.40	46.67 ± 6.10	− 2.522•	0.019	S
	Range	38–53	38–60			
Nasal	Mean ± SD	42.75 ± 5.75	44.79 ± 4.70	− 2.605•	0.016	S
	Range	35–53	36–51			
Temporal	Mean ± SD	44.17 ± 4.08	45.83 ± 4.88	− 2.234•	0.036	S
	Range	37–57	40–61			

*P*-value > 0.05: non significant; *P*-value < 0.05: significant; *P*-value < 0.01: highly significant

•Paired *t*-test

**Fig. 2:**
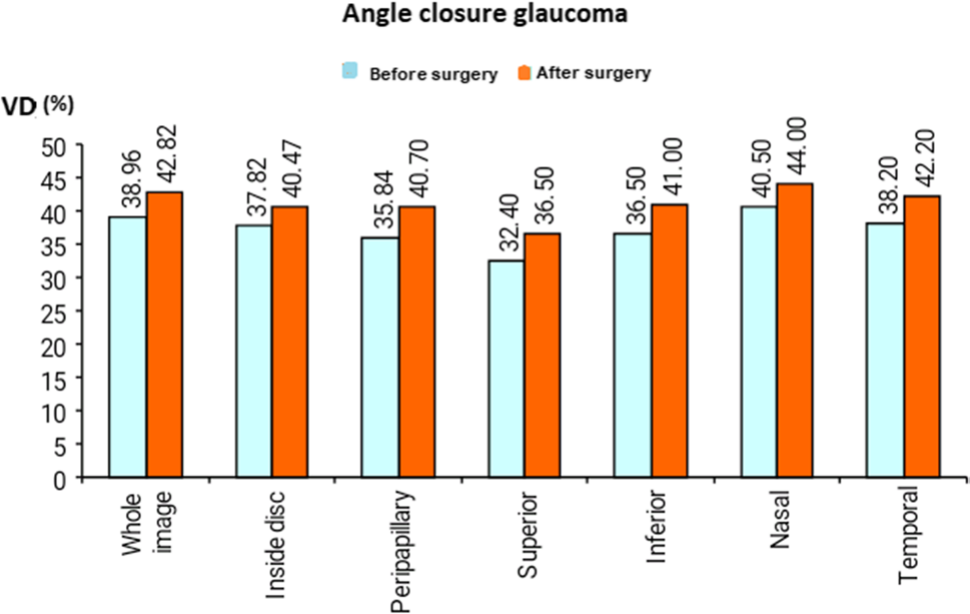
Comparison of optic disc vessel density (VD) before and after trabeculectomy in angle closure glaucoma (ACG)

**Fig. 3:**
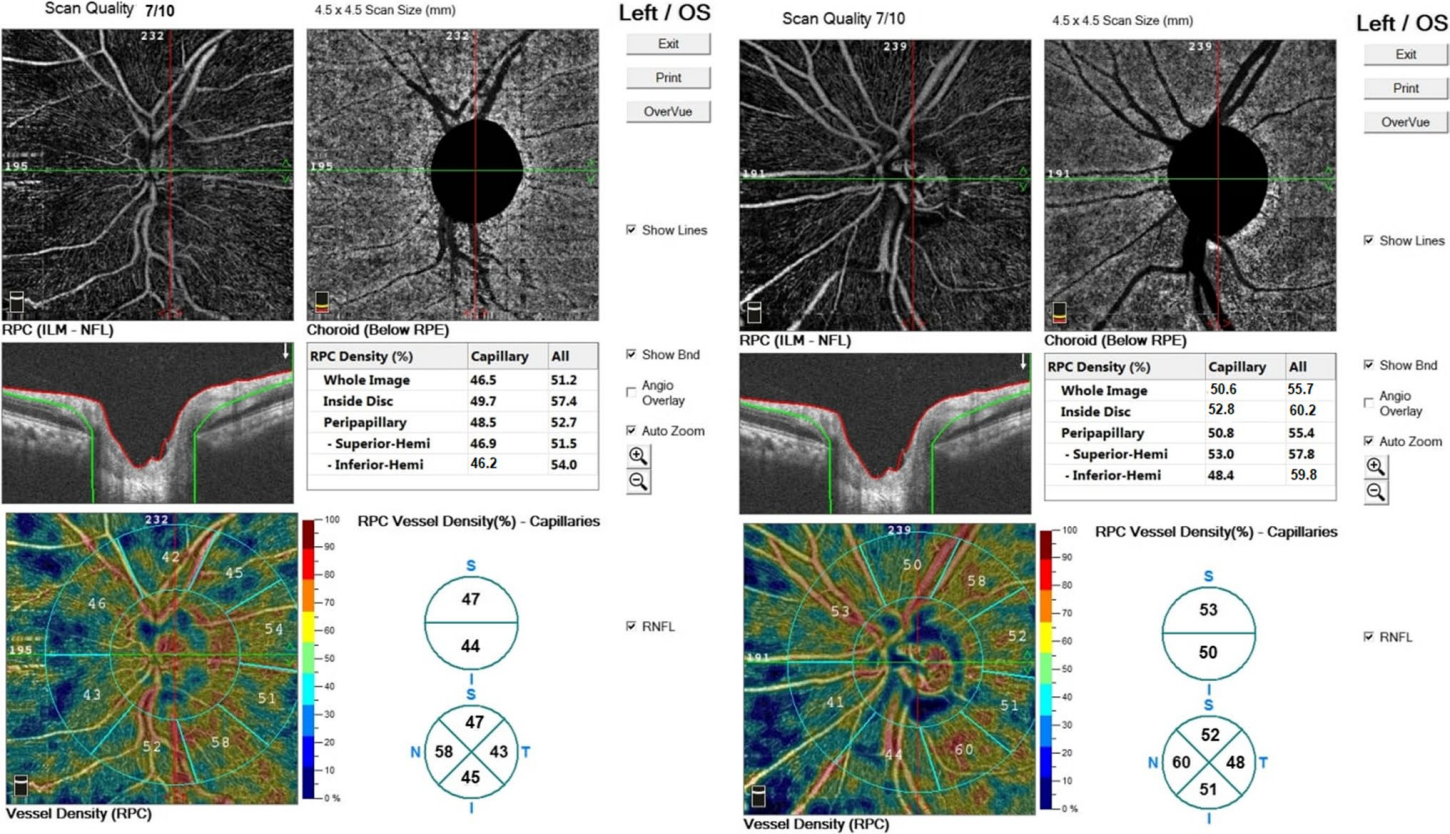
An increase in optic disc vessel density (VD) after trabeculectomy. The left image was before trabeculectomy. The right image was after trabeculectomy

As regards the change in VD after surgery in ACG patients, there was a statistically significant increase in optic disc VD in the whole image, radial peripapillary, inferior superior, temporal, and nasal quadrant in ACG after surgery (Figs. 2 and 3) (Table 4).

**Table 4:** The optic disc vessel density after trabeculectomy in angle-closure glaucoma

Vessel density %		Closed angle		Test value	*P*-value	Sig.
		Before surgery	After surgery			
Whole image	Mean ± SD	38.96 ± 5.53	42.82 ± 2.21	− 2.741•	0.023	S
	Range	35.4–51.2	40–45.8			
Inside disc	Mean ± SD	37.82 ± 6.33	40.47 ± 6.36	− 2.775•	0.022	S
	Range	29.7–48.4	32.6–53			
Peripapillary	Mean ± SD	35.84 ± 7.99	40.70 ± 8.66	− 3.073•	0.013	S
	Range	21.7–44.7	27.8–50.6			
Superior	Mean ± SD	32.40 ± 7.73	36.50 ± 8.00	− 3.086•	0.013	S
	Range	20–42	23–51			
Inferior	Mean ± SD	36.50 ± 7.96	41.00 ± 4.27	− 2.635•	0.027	S
	Range	21–47	34–45			
Nasal	Mean ± SD	40.50 ± 3.06	44.00 ± 2.83	− 3.217•	0.011	S
	Range	37–47	39–47			
Temporal	Mean ± SD	38.20 ± 5.55	42.20 ± 1.81	− 2.768•	0.022	S
	Range	32–46	40–45			

*P*-value > 0.05: non significant; *P*-value < 0.05: significant; *P*-value < 0.01: highly significant

•Paired *t*-test

**Fig. 4:**
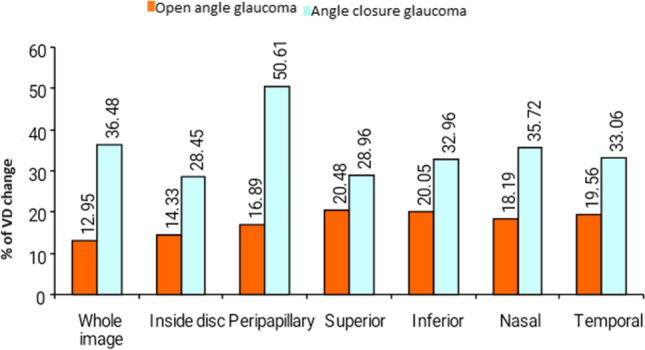
Comparison between open-angle glaucoma (OAG) and closed-angle glaucoma (ACG) as regards change in optic disc vessel density (VD) after trabeculectomy

With respect to the percentage of VD change following trabeculectomy surgery, there were highly significant differences between the two groups for the total image, radial peripapillary, inferior quadrant, and temporal quadrant VD. In addition, there were statistically significant differences in the VD of the superior and temporal quadrants. However, no significant change was detected in VD within the disc (Fig. 4 and Table 5).

**Table 5:** Comparison between vessel density change after trabeculectomy in open-angle and closed-angle glaucoma

% of VD change		Open angle	Closed angle	Test value	*P*-value	Sig.
		No. = 24	No. = 10			
Whole image	Mean ± SD	12.95 ± 4.29	36.48 ± 39.25	− 3.030 ≠	0.002	HS
	Range	8.02–23.24	12.99–110.38			
Inside disc	Mean ± SD	14.33 ± 3.05	28.45 ± 26.07	− 1.363 ≠	0.173	NS
	Range	9.89–19.79	9.5–77.1			
Peripapillary	Mean ± SD	16.89 ± 10.71	50.61 ± 61.03	− 3.787 ≠	< 0.001	HS
	Range	10.24–50.67	18.9–166.36			
Superior	Mean ± SD	20.48 ± 16.47	28.96 ± 12.13	− 2.575 ≠	0.010	S
	Range	10.2–70.97	16.22–50			
Inferior	Mean ± SD	20.05 ± 12.45	32.96 ± 15.91	− 3.030 ≠	0.002	HS
	Range	10–56.41	18.18–61.9			
Nasal	Mean ± SD	18.19 ± 9.14	35.72 ± 28.76	− 2.578 ≠	0.010	S
	Range	8.51–38.89	15.38–87.5			
Temporal	Mean ± SD	19.56 ± 14.58	33.06 ± 21.19	− 3.191 ≠	0.001	HS
	Range	12.77–64.86	19.44–73.08			

*P*-value > 0.05: non significant; *P*-value < 0.05: significant; *P*-value < 0.01: highly significant

≠ Mann–Whitney test

**Fig. 5:**
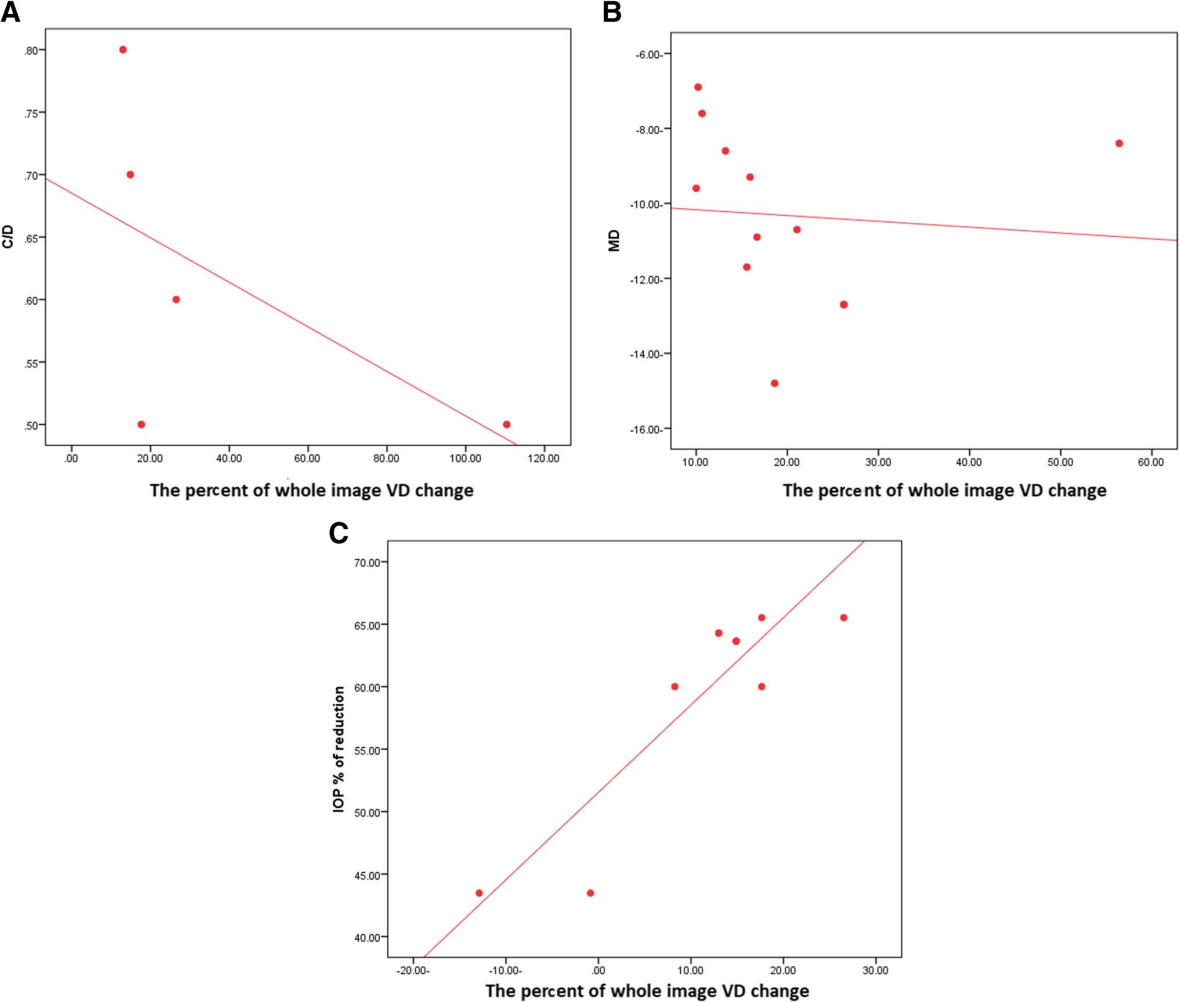
**A** A negative correlation between the change in whole image vessel density after surgery and C/D ratio in open-angle glaucoma. **B** A negative correlation between the change in whole image vessel density after surgery and mean deviation of the visual field (MD) in open-angle glaucoma. **C** A positive correlation between the change in whole image vessel density after surgery and present of IOP reduction in angle closure glaucoma

In OAG, there was a negative correlation between the whole image VD change and C/D with a *P*-value = 0.004. There was also a negative correlation between the whole image VD change and mean deviation in the visual field (MD) with a *P*-value = 0.016 (Fig. 5) (Table. 6).

**Table 6:** Correlation of vessel density change after subscleral trabeculectomy in open-angle and angle-closure glaucoma

	Whole image vessel density change													
	IOP pre		IOP post		IOP % of reduction		C/D		CCT		MD		Total RNFL	
	*r*	*P*-value	*r*	*P*-value	*r*	*P*-value	*r*	*P*-value	*r*	*P*-value	*r*	*P*-value	*r*	*P*-value
OAG	− 0.246	0.247	− 0.011	0.960	− 0.226	0.289	− 0.821**	**0.004**	− 0.401	0.052	− 0.488*	**0.016**	− 0.378	0.069
ACG	− 0.323	0.363	0.288	0.420	0.708*	**0.022**	− 0.013	0.972	0.174	0.631	0.242	0.500	0.571	0.084

*P*-value > 0.05: non significant; *P-value < 0.05: significant; **P-value < 0.01: highly significant

Spearman correlation coefficient

Regarding ACG, there was a positive correlation between the whole image VD change and the percentage of IOP reduction, with a *P*-value = 0.022 (Fig. 5) (Table 6).

## Discussion

In 2020, approximately 80 million people worldwide were estimated to have glaucoma. Glaucoma causes ocular morphological changes, including retinal ganglion cell apoptosis, peripapillary RNFL thinning, and intrapapillary nerve fiber loss. This results in the removal of the optic nerve head (ONH). Glaucoma patients exhibit both ocular and non-ocular vascular abnormalities. This results in vascular dysregulation, affecting the optic nerve and retina’s perfusion, which plays a crucial role in glaucoma progression [6].

There are two hypotheses for glaucoma: (a) mechanical theory: increased IOP compresses the optic nerve, and (b) vascular theory: dysregulation of ocular blood flow leads to ischemia which causes optic nerve damage [3].

OCT-A is an essential non-invasive technique. It plays a vital role in glaucoma follow-up, particularly in the advanced stages. This is due to the absence of the floor effect in OCT-A [3], which occurs when measuring RNFL thickness [3].

Currently, trabeculectomy is the most important surgical treatment for glaucoma. It is the most efficient surgical procedure for reducing IOP. Trabeculectomy is indicated in OAG patients with progressive optic nerve damage or visual field loss despite maximal medication dosage. In ACG, trabeculectomy is recommended if synechia exceeds 180° [7].

Some studies [3, 7–11] have reported a decrease in optic disc VD in patients with OAG than in the normal control population, which emphasizes the vascular theory of glaucoma.

In our study, the optic disc VD increased after lowering the IOP in OAG by trabeculectomy. This finding aligns with several studies [12–16] investigating the effect of lowering IOP by surgical methods. In addition, Holló [17], De Paula et al. [18], and Elsayed et al. [16] reported an increase in optic disc VD in patients with OAG after controlling IOP with medications.

However, Lommatzsch et al. [7], Güngör et al. [1], and Zéboulon et al. [4] reported a non significant change in optic disc VD following surgery to reduce IOP. The discrepancy in results may be due to age differences between patients. Our patients are significantly younger than others. This study’s mean age was 56.25 ± 11.37 years, whereas the mean ages in other studies were 66, 63.8 ± 5.97, and 61.1 ± 14.5 for Lommatzsch et al. [7], Güngör et al. [1], and Zéboulon et al. [4], respectively. The type of surgery may also lead to this discrepancy (Zéboulon et al. use non-penetrating glaucoma surgery). Moreover, the IOP reduction percentage may affect the VD change after surgery.

Few studies [2, 11, 19] have investigated the optic disc VD in ACG patients. They have reported decreased optic disc VD in those patients, consistent with our findings.

No prior studies have investigated the effect of IOP reduction in ACG patients on optic disc VD. Our study revealed an increase in optic disc VD after surgery in patients with ACG.

In this study, the improvement in optic disc VD may be due to the high percentage of IOP reduction after surgery in both OAG and ACG. The percentages of IOP reduction were 58.24 ± 5.25% and 59.38 ± 8.60, respectively, which has led to relieving the mechanical pressure over optic disc blood vessels.

Comparing the effect of IOP reduction on optic disc VD after glaucoma surgery in OAG and ACG, ACG demonstrates more significant improvement than OAG. We have two hypotheses for this result. The first is that the higher IOP in ACG has a greater impact on the blood vessels of the optic nerve than in OAG. Consequently, surgical reduction of IOP results in a greater improvement in optic disc VD in ACG than in OAG. Second, there may be dysregulation of blood vessels in OAG, resulting in less improvement of optic disc VD after IOP reduction. This dysregulation may be due to the older age of patients with OAG (OAG occurs at an older age than ACG). Furthermore, the late presentation of OAG and the long duration of the disease may affect blood vessel dysregulation and make the change in blood vessels permanent. In contrast, ACG is presented early, with early surgical interference in most cases. This hypothesis is supported by the negative correlation between both the C/D ratio and MD of the visual field with the percent of whole image VD change. This suggests that the alterations in VD decrease as OAG disease severity increases. Moreover, the thinner RNFL in ACG with more severe disease may reflect the difference in blood vessel autoregulation and sensitivity to pressure fluctuations. The absence of a correlation between the change in VD and RNFL in both OAG and ACG may not support this hypothesis.

No study has been discovered comparing OAG and ACG with the change in optic disc VD after surgery.

These results are significant since they highlight the role of the vascular theory in glaucoma (both OAG and ACG). Therefore, we may attempt to improve the optic disc perfusion in ways besides lowering IOP. Moreover, optic disc VD can be used as an indicator of the disease’s control (good optic disc microvasculature indicates effective disease control). Therefore, OCT-A can be used for follow-up glaucoma patients. More research is required to investigate the role of OCT-A in glaucoma patient follow-up and to implement the use of optic disc VD as a secondary tool in glaucoma patient follow-up. This study has some limitations. First, there was a small sample size. Second, the period of follow-up was brief. It is necessary to conduct studies with large sample sizes and lengthy durations.

In conclusion, the optic disc VD improved after the reduction of IOP by surgery in both OAG and ACG. However, AGC demonstrated a more significant improvement than OAG.
